# Hue-shifted monomeric variants of *Clavularia *cyan fluorescent protein: identification of the molecular determinants of color and applications in fluorescence imaging

**DOI:** 10.1186/1741-7007-6-13

**Published:** 2008-03-06

**Authors:** Hui-wang Ai, Scott G Olenych, Peter Wong, Michael W Davidson, Robert E Campbell

**Affiliations:** 1 University of Alberta, Department of Chemistry, Edmonton, Alberta, T6G 2G2 Canada; 2 National High Magnetic Field Laboratory and Department of Biological Science, Florida State University, 1800 East Paul Dirac Drive, Tallahassee, FL 32310, USA

## Abstract

**Background:**

In the 15 years that have passed since the cloning of *Aequorea victoria *green fluorescent protein (avGFP), the expanding set of fluorescent protein (FP) variants has become entrenched as an indispensable toolkit for cell biology research. One of the latest additions to the toolkit is monomeric teal FP (mTFP1), a bright and photostable FP derived from *Clavularia *cyan FP. To gain insight into the molecular basis for the blue-shifted fluorescence emission we undertook a mutagenesis-based study of residues in the immediate environment of the chromophore. We also employed site-directed and random mutagenesis in combination with library screening to create new hues of mTFP1-derived variants with wavelength-shifted excitation and emission spectra.

**Results:**

Our results demonstrate that the protein-chromophore interactions responsible for blue-shifting the absorbance and emission maxima of mTFP1 operate independently of the chromophore structure. This conclusion is supported by the observation that the Tyr67Trp and Tyr67His mutants of mTFP1 retain a blue-shifted fluorescence emission relative to their avGFP counterparts (that is, Tyr66Trp and Tyr66His). Based on previous work with close homologs, His197 and His163 are likely to be the residues with the greatest contribution towards blue-shifting the fluorescence emission. Indeed we have identified the substitutions His163Met and Thr73Ala that abolish or disrupt the interactions of these residues with the chromophore. The mTFP1-Thr73Ala/His163Met double mutant has an emission peak that is 23 nm red-shifted from that of mTFP1 itself. Directed evolution of this double mutant resulted in the development of mWasabi, a new green fluorescing protein that offers certain advantages over enhanced avGFP (EGFP). To assess the usefulness of mTFP1 and mWasabi in live cell imaging applications, we constructed and imaged more than 20 different fusion proteins.

**Conclusion:**

Based on the results of our mutagenesis study, we conclude that the two histidine residues in close proximity to the chromophore are approximately equal determinants of the blue-shifted fluorescence emission of mTFP1. With respect to live cell imaging applications, the mTFP1-derived mWasabi should be particularly useful in two-color imaging in conjunction with a Sapphire-type variant or as a fluorescence resonance energy transfer acceptor with a blue FP donor. In all fusions attempted, both mTFP1 and mWasabi give patterns of fluorescent localization indistinguishable from that of well-established avGFP variants.

## Background

In 1992 the scientific community was gifted with a research tool that profoundly and irreversibly changed the way researchers approach the study of protein function in live cells [1]. The tool was, of course, the gene encoding the *Aequorea victoria *green fluorescent protein (avGFP) [2]. Soon after the first demonstrations of functional expression of the gene encoding avGFP in organisms other than jellyfish [3,4], published reports of the use of fluorescent proteins (FPs) for microscopy applications 'took off' [5]. Since that time, the impact of FPs on the life sciences has continued to increase with each passing year and this growth shows no signs of slowing [5]. One important driving force behind the ever-increasing popularity of FPs is the fact that researchers continue to create FPs with wavelength-shifted absorbance and/or emission wavelengths and/or improved or novel properties (for example, increased brightness, improved photostability or photoactivation) [6,7]. Improved FPs facilitate life science research by minimizing technical hurdles that otherwise complicate their use in imaging applications. For example, FPs with improved photostability enable time-lapse imaging over greater durations. FPs with novel properties can inspire the development of entirely new applications that would otherwise be impractical or even impossible. This has certainly been the case with photoactivatable FPs that have enabled cellular imaging at resolutions beyond the diffraction limit [8].

**Figure 1:**
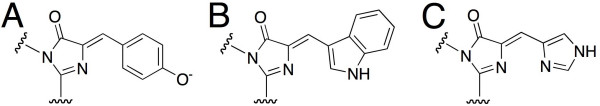
**Chromophore structures of mTFP1 and its hue-shifted variants**. (A) The chromophore structure shared by EGFP, mTFP1 and mWasabi. (B) The chromophore structure shared by ECFP and the mTFP1-Y67W variant. (C) The chromophore structure shared by EBFP and the mTFP1-Y67H variant.

**Figure 2:**
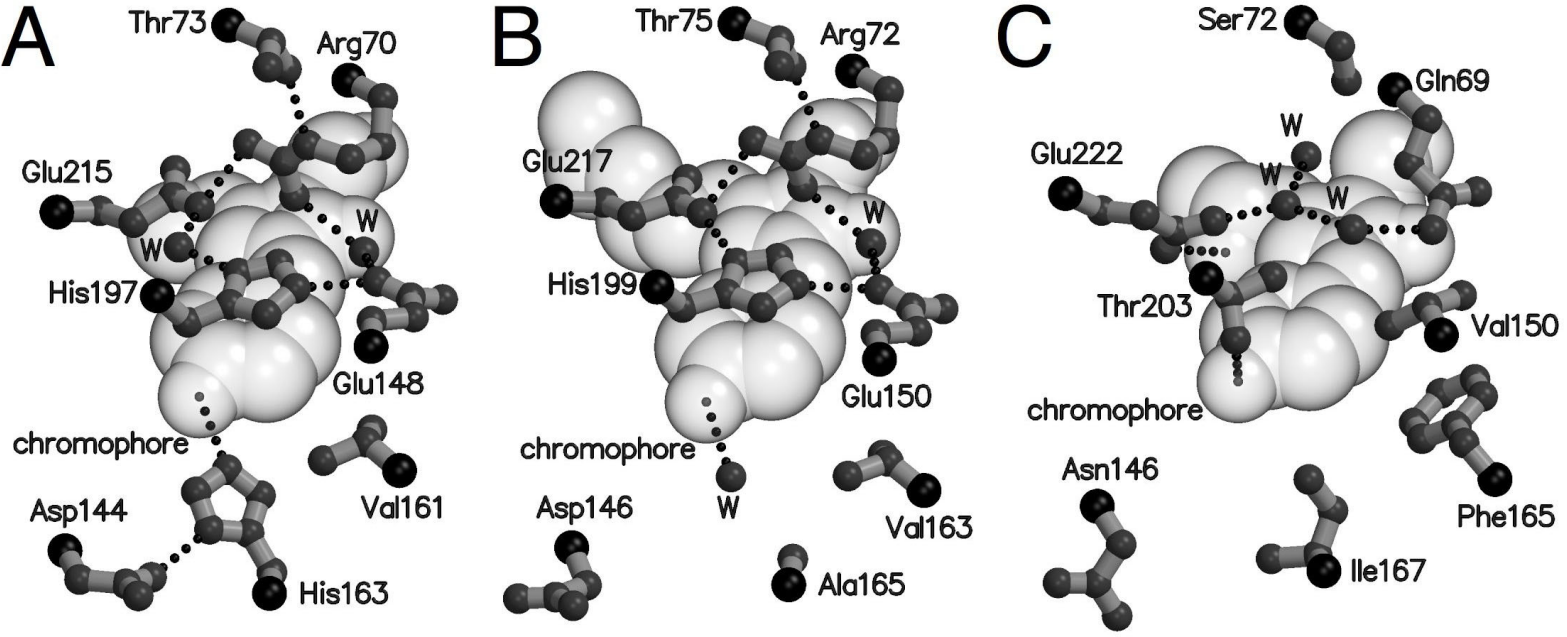
**The chromophore environment of mTFP1, amFP486 and avGFP-S65T**. (A) The chromophore of mTFP1 (Protein data bank (PDB) code 2HQK) in space filling representation [19]. The side chains of residues that are discussed in the text and that are in close proximity to the chromophore are shown in ball-and-stick. Hydrogen bonds are indicated with black dotted lines. Cα for each residue is represented as a black sphere. Atoms labeled 'W' are ordered water molecules. (B) The chromophore environment of amFP486 showing the residues that are structurally aligned with the residues represented in (A) (PDB code 2A46) [20]. (C) The chromophore environment of avGFP-S65T (and EGFP) showing the residues that structurally align with those represented in (A) (PDB code 1EMA) [26]. avGFP-S65T and EGFP differ only by the Phe64Leu mutation which does not significantly modify the conformation of any residues shown in this figure.

**Figure 3:**
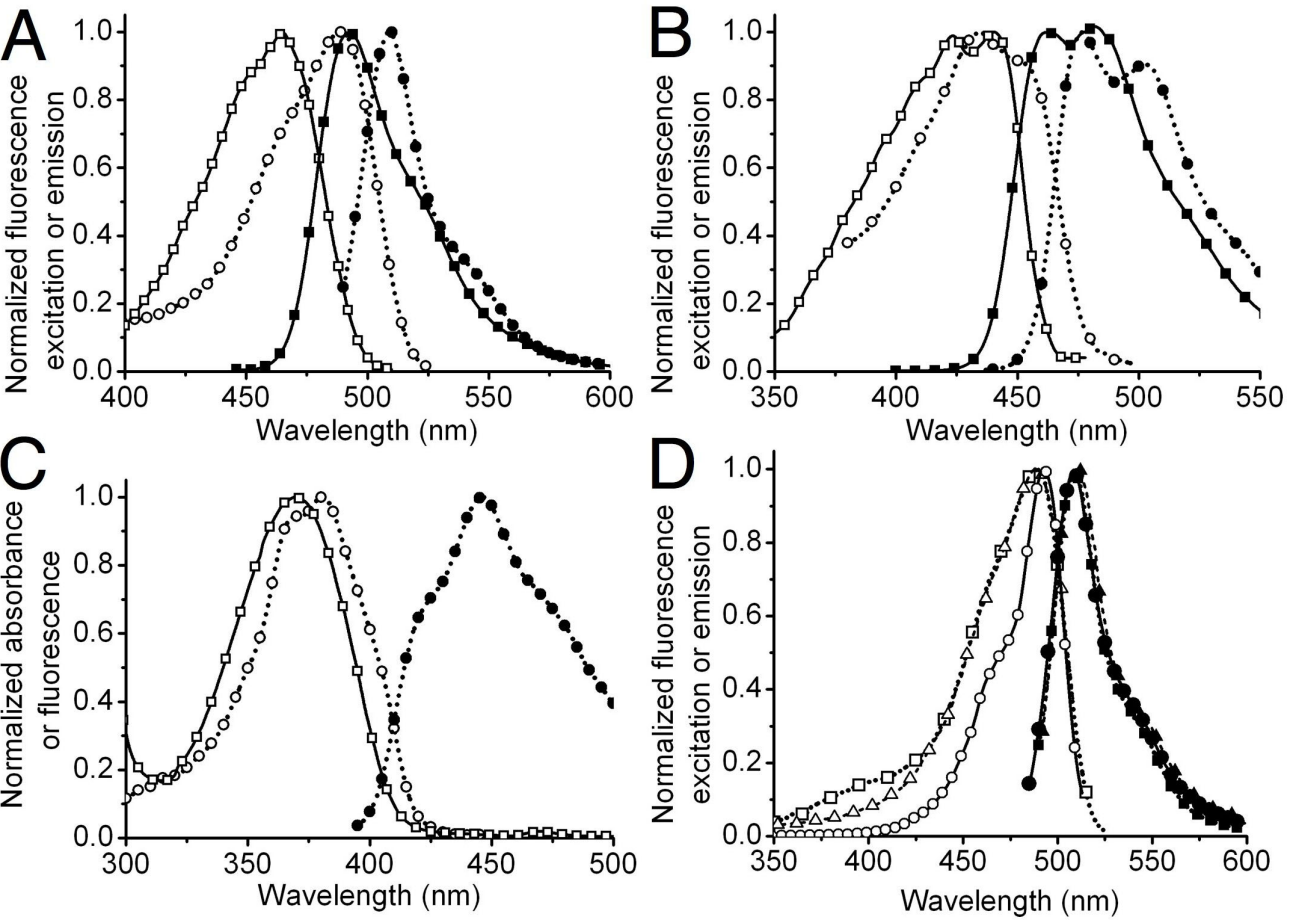
**Spectra of hue-shifted variants of mTFP1**. (A) Excitation (open symbols) and emission (filled symbols) spectra of EGFP (circle) and mTFP1 (square). (B) Excitation (open symbols) and emission (filled symbols) spectra of ECFP (circle) and mTFP1-Y67W (square). (C) Excitation (open symbols) and emission (filled symbols) spectra of EBFP (circle) and the absorbance (open symbols) spectrum of the nonfluorescent mTFP1-Y67H variant (square). (D) Excitation (open symbols) and emission (filled symbols) spectra of mWasabi (circle), EGFP (square) and Emerald (triangle). Spectra were collected at 1 nm steps, but only every fifth data point is shown for clarity.

The availability of engineered avGFP variants with altered color, where color refers to the absorbance and/or fluorescence emission spectral profiles, has been a boon to life science research. Access to a wide ranging FP color palette has allowed researchers to simultaneously track multiple proteins or use fluorescence resonance energy transfer (FRET) to detect protein-protein interactions in a live cell [9]. Fortunately, avGFP has been a fertile source of new colors of FPs. The main classes of color variants derived from avGFP include those that are blue fluorescent [10], cyan fluorescent [11], cyan-excitable green fluorescent [12], UV-excitable green fluorescent [13] and yellow fluorescent [14]. Coral is also an abundant source of FPs [15,16] and in recent years this treasure trove, which includes variants with fluorescent hues ranging from cyan to far-red, has yielded a number of exciting new variants [6,17,18]. For example, we recently described [19] the engineering of a codon optimized and monomeric version of cFP484, a tetrameric cyan FP (CFP) from *Clavularia *coral [15]. The resulting protein, known as monomeric teal FP (mTFP1), has an anionic tyrosine-derived chromophore that is chemically identical to that of enhanced avGFP (EGFP; see Figure 1A). However, the absorbance and fluorescence emission maxima of mTFP1 (emission maximum = 492 nm) are blue shifted by about 15 nm relative to EGFP (emission maximum = 507 nm) owing to numerous amino acid differences in the chromophore-containing cavity [19,20] (compare Figures 2A and 2C and see Figure 3A). We have demonstrated that mTFP1 is a favorable alternative to avGFP-derived CFPs with tryptophan-derived chromophores such as enhanced CFP (ECFP) or Cerulean [11]. The specific advantages of mTFP1 include a narrower and single-peaked emission spectrum, improved brightness and improved photostability [19].

In this work we report on our efforts to engineer a series of new colors of mTFP1-derived FPs through the use of site-directed mutagenesis and random mutagenesis with library screening. This work has provided important insight into the amino acid determinants of color in mTFP1. In addition we have undertaken a thorough assessment of mTFP1 fusion proteins to determine whether such constructs exhibit their expected pattern of subcellular localization. Together these new results further establish mTFP1, and its suitably optimized hue-shifted variants, as useful new additions to the toolkit of FPs for cell biology research.

## Results and discussion

### Blue-shifted variants of mTFP1

A series of computational studies have provided support for the idea that there is a partial transfer of charge from the phenolate moiety to the imidazolinone moiety (Figure 1A) in the excited state of the avGFP anion [21,22]. Since the phenolate is more electron rich in the ground state than in the excited state, factors that contribute to charge stabilization will tend to increase the energy barrier for charge transfer and shift the excitation and emission peaks to higher-energy wavelengths (that is, towards the blue). The crystal structures of mTFP1 [19] and amFP486 [20] (a tetrameric cyan-fluorescing FP from *Anemonia majano*) revealed that these homologous blue-shifted FPs both have structurally analogous histidine imidazoles, His197 of mTFP1 and His199 of amFP486, stacked against the phenolate ring of the chromophore (Figure 2A and 2B). Owing to the involvement of the imidazole in a quadrupole salt bridge network (with Glu148, Glu215 and Arg70 in mTFP1; Glu150, Glu217 and Arg72 in amFP486) it is likely to have significant cationic character. A simple electrostatic interpretation of the imidazole-chromophore interaction might therefore suggest that this cationic character is helping to stabilize anionic character on the phenolate ring. Other mutagenesis-based studies have provided support for the idea that the side chain of the residue aligning with residue His163 of mTFP1 (Figure 2A), or a buried water molecule that occupies the cavity when the side chain is small (as is the case of Ala165 in amFP486 as shown in Figure 2B), also has an important role in stabilizing anionic character on the phenolate ring [23]. Henderson and Remington have proposed that the electrostatic interaction with His199 is of greater significance than the interaction with the water molecule in the residue 165 side-chain cavity for causing the blue-shifted emission of the amFP486 chromophore [20]. The relative importance of His197 and His163 with respect to the blue-shift of the mTFP1 chromophore has not been investigated.

We reasoned that if this electrostatic-based mechanism for 'fine tuning' of the emission wavelength is indeed operative in mTFP1, variants with alternative chromophore structures, should also be blue-shifted relative to their avGFP analogs. Two qualifications are that formation of the excited state still involves charge transfer to the imidazolinone ring and that significant repacking of the side chains lining the chromophore-containing cavity does not occur with the new chromophore structure. To investigate whether this mechanism for blue-shifting the fluorescence could be translated to alternative chromophore structures, we created the Tyr67Trp and Tyr67His mutants of mTFP1. The chromophore structures of mTFP1-Y67W and mTFP1-Y67H are chemically identical to that of avGFP-derived ECFP and EBFP, respectively (Figures 1B and 1C). Accordingly, we expected that the absorbance and fluorescence emission maxima of mTFP1-Y67W and ECFP (and mTFP1-Y67H and enhanced blue FP (EBFP)) would be similar but not necessarily identical. If differences between the spectra of the two proteins were observed, they must be attributable to the effect of the protein environment on the chromophore.

Measuring the absorbance and emission spectra of purified mTFP1-Y67W revealed that this protein is fluorescent, exhibits the typical double-humped peaks associated with a tryptophan-derived chromophore and is blue-shifted relative to ECFP (Figure 3B). Considering the mean wavelength at half maximum intensity, the excitation and emission peaks are shifted by 13 and 13.5 nm, respectively. This result contrasts with a previous report in which it was demonstrated that the analogous substitution in amFP486 results in a protein that is not blue-shifted relative to avGFP-derived CFP [23]. The purified mTFP1-Y67H variant exhibited no significant fluorescence, but did have a strong absorbance peak that was blue-shifted by 10.5 nm (mean wavelength at half maximum intensity relative to avGFP-derived EBFP (Figure 3C).

These results demonstrate that the protein-chromophore interactions responsible for blue shifting the absorbance and emission maxima (that is raising the energy of the excited state) of mTFP1 are not entirely dependent on the presence of a tyrosine-derived chromophore. In the crystal structure of mTFP1, the imidazole of His163 is observed to be making a hydrogen bond with the phenolate oxygen of the chromophore (Figure 2A). An analogous interaction is not possible in the mTFP1-Y67H or mTFP1-Y67W variants. In contrast, the close stacking of the His197 imidazole against the chromophore phenolate is an interaction that could be preserved in the mTFP1-Y67W or mTFP1-Y67H variants. We conclude that the hydrogen bond with His163 is not significant with respect to the blue-shift of mTFP1 and it is either the close stacking of the His197 imidazole and/or a hydrogen bond-independent electrostatic effect of His163 that is responsible for the blue-shift. Based on the interaction of His163 with the carboxylate of Asp144 (Figure 2A), it is plausible that the imidazole could have significant cationic character.

### Red shifted variants of mTFP1

A reasonable approach to dissecting the relative importance of His163 and His197 in blue-shifting mTFP1 fluorescence is to examine variants in which the identity of one residue is changed through the use of site-directed mutagenesis. It has previously been shown that His199 of amFP486, which is structurally analogous to His197 of mTFP1, is stacked against the chromophore and has multiple critical roles that dictate the spectroscopic properties (Figure 2B) [20]. We expected that, in the absence of high-resolution crystal structures, interpretation of the effects of mutation at this position would pose a significant challenge. We opted instead to focus on His163 since it is not strictly conserved between the natural cyan-fluorescing proteins and thus less likely to have multiple critical roles. We performed saturation mutagenesis of mTFP1 at position 163 and screened the library using a colony-based fluorescence imaging system. Screening revealed that the library contained both brightly cyan-fluorescing and green-fluorescing members. DNA sequencing revealed that the bright cyan-fluorescing members of the library had, as expected, a histidine at position 163 and were thus identical to mTFP1. The brightest green-fluorescing member had a methionine at position 163 and a fluorescence emission maximum at 503 nm (Table 1). The fact that the emission maximum of mTFP1-H163M is 11 nm red-shifted from that of mTFP1 provides strong support for His163 contributing to the blue-shift of the mTFP1 chromophore by an electrostatic mechanism. As to why methionine, as opposed to some other amino acid, was identified as the best replacement for His163, we can suggest two possible reasons. The first is that the methionine side chain could simply be the best steric fit in the cavity formerly filled by the side chain of His163. The second is that the sulfur atom of the methionine side chain may be participating in sulfur-aromatic interactions with the chromophore. Such interactions have previously been used to explain improvements in the extinction coefficient in avGFP variants [24].

**Table 1:** Properties of hue-shifted mTFP1 variants

	Excitation maximum (nm)	Emission maximum (nm)	ε(mM^-1 ^cm^-1^)	Φ	Brightness^a ^(mM^-1 ^cm^-1^)	pKa	Photostability^b^
mTFP1-Y67W	424/440^c^	461/482^c^	13	0.02	0.3	ND^d^	ND
mTFP1-Y67H	369^e^	NA^f^	7	NA	NA	ND	NA
G1	487	503	43	0.60	26	ND	ND
G2	487	503	60	0.65	39	ND	65
G3	498	515	70	0.70	49	ND	5.5
mWasabi	493	509	70	0.80	56	6.5	93

mTFP1^g^	462	492	64	0.85	54	4.3	110
EGFP^h^	488	507	56	0.60	34	6.0	174

^a^Product of extinction coefficient and quantum yield.

^b^Time in seconds to bleach from an initial emission rate of 1000 to 500 photons s^-1 ^molecule^-1^.

^c^Values for both 'humps' are provided.

^d^ND, not determined.

^e^This value is the absorption maximum. No significant fluorescence was detected for mTFP1-Y67H.

^f^NA, not applicable.

^g^Values from [19].

^h^Values from [7].

So is His163 solely responsible for the blue-shift of mTFP1 or does His197 also play a role? That is, does the emission maximum of 503 nm for mTFP1-H163M represent an appropriate 'reference' state for this particular chromophore structure when located in this particular chromophore cavity and in the complete absence of any blue-shifting effects? Previous work suggests that the answer to the latter question is probably no, and the emission maximum of the reference state is more likely to be approximately 515 nm. One piece of evidence supporting this suggestion is that the emission maximum of amFP486-H199T is 515 nm [20]. This variant has essentially an identical chromophore cavity to mTFP1, with the obvious exception of the His199Thr replacement. The second piece of evidence in support of this suggestion is that the avGFP-T203H mutant has a fluorescence emission at 517 nm when excited at 475 nm (see [25]). Residue Thr203 of avGFP [26] is structurally aligned with His197 of mTFP1 (compare Figures 2A and 2C), and thus avGFP-T203H likely has an imidazole-chromophore stacking interaction similar to that of mTFP1 [20]. However, unlike the positively charged imidazole of His197 in mTFP1, the imidazole of His203 in avGFP-T203H is expected to be in the neutral-charge state.

In a later section we describe the discovery of the Thr73Ala substitution that red-shifted the fluorescence of mTFP1-K139E/H163M from 503 to 515 nm. However, it is appropriate to discuss the implications of this fortunate finding in the current context. In the crystal structure of mTFP1, the hydroxyl group of Thr73 is hydrogen-bonded to the guanidium group of Arg70: a key participant of the quadrupole salt-bridge network responsible for maintaining the imidazole of His197 in the positively charged state (Figure 2A). We propose that the loss of the Thr73-Arg70 hydrogen bond in the Thr73Ala mutant perturbs the salt-bridge network such that the cationic character of His197 imidazole is greatly diminished or abolished. Accordingly, the Thr73Ala mutant effectively separates the electrostatic role of His197 from its additional roles in maintaining the chromophore environment and reveals that the electrostatic effect of this residue accounts for a blue-shift of at least 12 nm.

Our mutagenesis-based study supports the conclusion that His163 and His197 act in concert to blue-shift the fluorescence emission of the mTFP1 chromophore through an electrostatic mechanism. The contribution of both residues is effectively identical with 11 and 12 nm of blue-shift attributed to His163 and His197, respectively. This result is essentially consistent with previous studies of amFP486 which have suggested complementary roles for His199 and the water molecule adjacent to Ala165 in achieving the blue-shift [20,23]. The crystallographic and mutational study by Henderson and Remington supports the conclusion that the water molecule adjacent to Ala165 has a less significant contribution than His199 [20]. In mTFP1 the (plausibly) cationic imidazole group of His163 could contribute a significant amount of electrostatic stabilization to electron density on the phenolate ring. A similar interaction is not possible in amFP486 since a lone water molecule sits in the location occupied by the His163 imidazole of mTFP1. A caveat is that the electron density modeled as a water in the amFP486 structure could actually be a sodium ion or other cation (S J Remington, personal communication), in which case an electrostatic contribution would not be unreasonable.

### Directed evolution of the red shifted variant of mTFP1

Intrigued by the high apparent fluorescent brightness of mTFP1-H163M (hereafter designated G1), we subjected this template to directed evolution in an effort create a new GFP variant that could potentially be useful for live cell imaging. Error-prone polymerase chain reaction (PCR) was used to create libraries of genetic variants, the gene libraries were expressed in *Escherichia coli*, and colonies were screened for bright green fluorescence. The brightest green fluorescent colony identified in the first round of screening was found to express a G1 variant with the additional mutation Lys139Met (designated G2). This variant was used as the template for a second round of library construction and screening. The brightest variant identified in the second round was found to be mTFP1-T73A/K139M/H163M (designated G3). It is interesting to note that the Thr73Ala mutation present in G3 is structurally aligned with the Ser72Ala mutation that has been reported to improve the brightness of avGFP variants [27]. No further improvements were identified during a third round of screening of randomly mutated variants based on the G3 template. *In vitro *characterization (Table 1) of the purified green fluorescing variants revealed that relative fluorescent brightness to be 1, 1.5 and 1.9 for G1, G2 and G3, respectively. While both G1 and G2 had fluorescence maxima at 503 nm, G3 was further red-shifted to 515 nm. The implications of this result have been discussed above.

Further investigation of the G2 and G3 variants revealed that the dimmer G2 was 11.8-fold more photostable than the brighter G3 variant. In our experience mutations that improve fluorescent brightness are much more readily identified than mutants that improve photostability. For this reason we forsook the brighter G3 variant and continued optimization based on the G2 template. Saturation mutagenesis at three positions chosen based on their proximity to the chromophore (Ala66, Val161 and Ile199) resulted in the identification of a further improved variant containing the Ala66Ser substitution. A subsequent round of random mutagenesis resulted in the identification of the Ser216Ile substitution. Additional rounds of random mutagenesis yielded no further improvements. The end product of this process is a brightly fluorescent GFP (emission maximum = 509 nm) that is equivalent to mTFP1-A66S/K139E/H163M/S216I and has been designated mWasabi. The fluorescence emission maximum of mWasabi is intermediate between that of G1 and G3, suggesting that there has been a perturbation of the salt-bridge network. It has been previously reported that avGFP with a Ser at residue 65 is 5 nm red-shifted from avGFP with an Ala at residue 65 (see [12]). As observed in the avGFP-S65T structure (Figure 2C), the hydroxyl group of the Ser at residue 66 of mWasabi could potentially form a new hydrogen bond with Glu215 and partially disrupt its ability to contribute to the critical salt-bridge network.

*In vitro *characterization of mWasabi (Table 1) revealed that it is 1.6-fold brighter than EGFP, making it one of the brightest and most photostable FPs currently available [7]. Another notable feature of mWasabi is its very narrow excitation and emission peaks (Figure 3D) that are reminiscent of the spectrum of *Renilla *GFP [28] and monomeric Azami-Green [29]. Narrower peaks allow for more efficient excitation and gathering of emission when used in combination with bandpass filters, and reduce the degree of bleed-through in multicolor imaging.

### Two-color imaging with mWasabi and Sapphire

**Figure 4:**
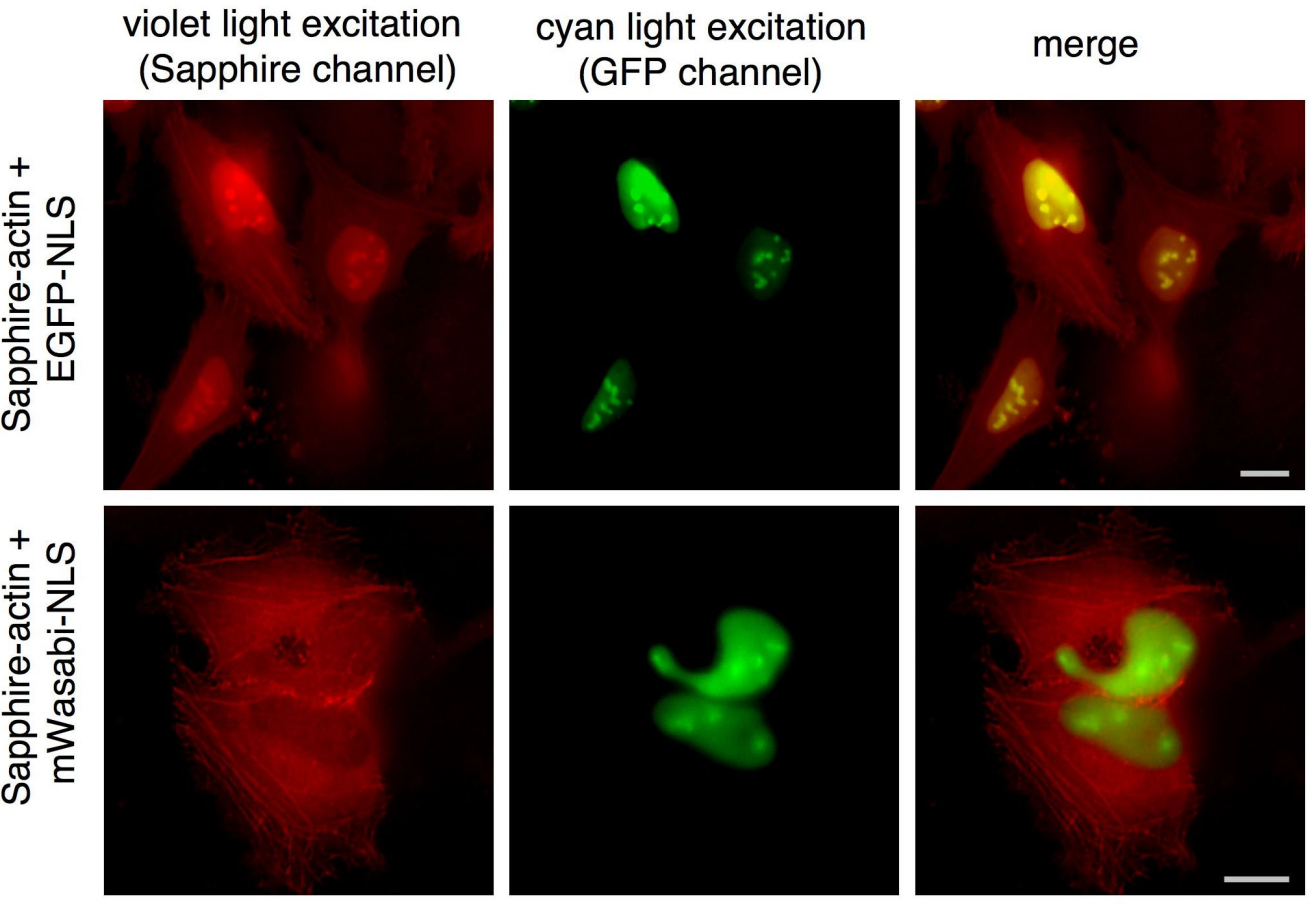
**Two-color imaging with Sapphire/EGFP**. Shown in the upper row of panels are HeLa cells that have been transfected with plasmids for expression of both Sapphire-actin and EGFP-NLS. Shown in the lower row of panels are identically treated HeLa cells expressing Sapphire-actin and mWasabi-NLS. Owing to the residual excitation of EGFP at 400 nm, the nucleus fluoresces brightly in the Sapphire emission channel in the top row of panels. In contrast, mWasabi is not significantly excited at 400 nm and thus the nucleus is much dimmer than the actin filaments in the bottom row of panels. Scale bars represent 10 μm.

EGFP and its descendents have their major absorption peaks at around 488 nm (see [30-32]). However, owing to both the breadth of this peak and the fact that in some variants a significant fraction of the protein exists as the UV-excitable neutral chromophore, EGFP and related variants are efficiently excited with violet light (~400 nm; see Figure 3D). This residual excitation with 400 nm light can unnecessarily complicate multicolor imaging in combination with a Sapphire-type variant [2,13,33] or fluorescence resonance energy transfer (FRET) experiments with a blue FP (BFP) donor [14,34]. In terms of fluorescence emission, mWasabi, EGFP and Emerald have almost identical emission peak shapes (Figure 3D). In contrast, the differences in their excitation spectra are pronounced, with mWasabi showing almost no excitable component below 410 nm. This suggested to us that mWasabi would be superior to EGFP for use in two-color imaging with Sapphire. To test this proposal, mWasabi and EGFP were fused with a nuclear localization signal (NLS) and separately co-expressed with Sapphire-β-actin in HeLa cells. As shown in Figure 4, exciting Sapphire with a typical 375–415 nm bandpass excitation filter resulted in significant EGFP fluorescence as observed in the cell nucleus. In contrast, no significant fluorescence was observed for mWasabi in the cell nucleus when Sapphire was imaged under identical conditions. This result demonstrates that mWasabi is particularly well suited for multicolor imaging in combination with fluorophores that are excitable with violet light.

### Imaging of mTFP1 and mWasabi fusion proteins

Both mTFP1 and its green-emitting progeny, mWasabi, are the products of an extensive process of protein engineering and directed evolution. During the development of these proteins, substantial effort was expended to find variants with the desired color, high fluorescent brightness, high folding and maturation efficiency and high photostability. Our *in vitro *characterization of these proteins confirms that we were indeed successful in engineering proteins with the desired properties. However, the ultimate goal of this research is not to simply develop new FPs, but rather to develop FPs that will be useful tools for fluorescence imaging in living cells. To be generally useful for live cell imaging, a FP should retain its favorable properties either when fused to a variety of proteins or when targeted to a variety of subcellular compartments. In addition, the FP should not perturb the normal localization or biological function of the protein to which it is genetically fused. Such a perturbation can be caused by oligomerization of the FP: a problem that should not be relevant to monomeric FPs such as mTFP1 and mWasabi.

**Figure 5:**
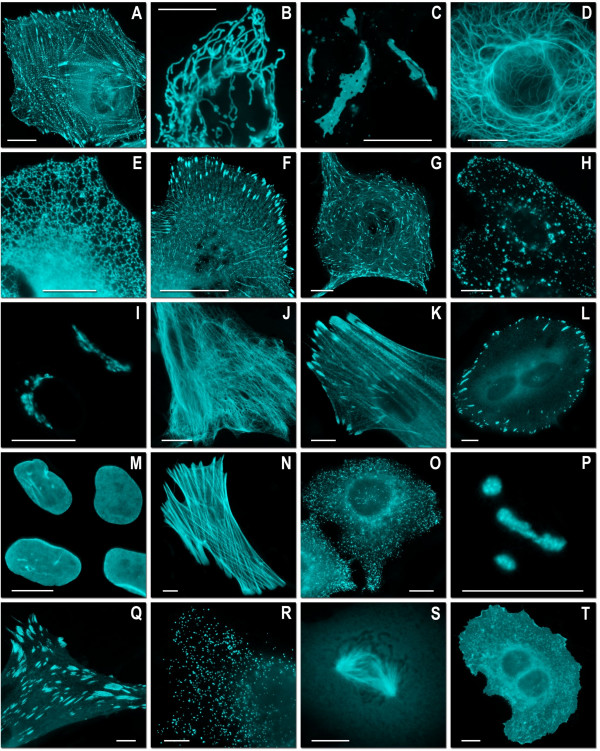
**Fluorescence imaging of mTFP1 fusion constructs**. (A)-(K) N-terminal fusion constructs; for each fusion protein the linker amino acid length is indicated after the name of the targeted organelle or fusion protein: (A) mTFP1-α-actinin-19 (human non-muscle); (B) mTFP1-mitochondria-7 (human cytochrome C oxidase subunit VIII); (C) mTFP1-Cx43-7 (rat α-1 connexin-43); (D) mTFP1-keratin-17 (human cytokeratin 18); (E) mTFP1-endoplasmic reticulum-3 (calreticulin signal sequence (51 nucleotides) and KDEL retention sequence); (F) mTFP1-paxillin-22 (chicken); (G) mTFP1-EB3-7 (human microtubule-associated protein; RP/EB family); (H) mTFP1-lysosomes-20 (rat lysosomal membrane glycoprotein 1); (I) mTFP1-golgi-7 (N-terminal 81 amino acids of human β-1,4-galactosyltransferase); (J) mTFP1-vimentin-7 (human); (K) mTFP1-zyxin-7 (human). (L)-(T) C-terminal fusion constructs: (L) mTFP1-focal adhesion kinase-5 (chicken protein tyrosine kinase 2); (M) mTFP1-lamin B1–10 (human); (N) mTFP1-β-actin-7; (O) mTFP1-clathrin light chain-15 (human); (P) mTFP1-fibrillarin-7 (human); (Q) mTFP1-vinculin-23 (human); (R) mTFP1-peroxisomes-2 (peroximal targeting signal 1; PTS1); (S)mTFP1-β-tubulin-6 (human); (T) mTFP1-farnesyl-5 (20-amino acid farnesylation signal from c-Ha-Ras). The cell line used for expressing mTFP1 fusion vectors was gray fox lung fibroblast cells (FoLu) in panels (A), (G), (K), (N) and Q) and human cervical adenocarcinoma cells (HeLa) in the remaining panels. Scale bars represent 10 μm.

**Figure 6:**
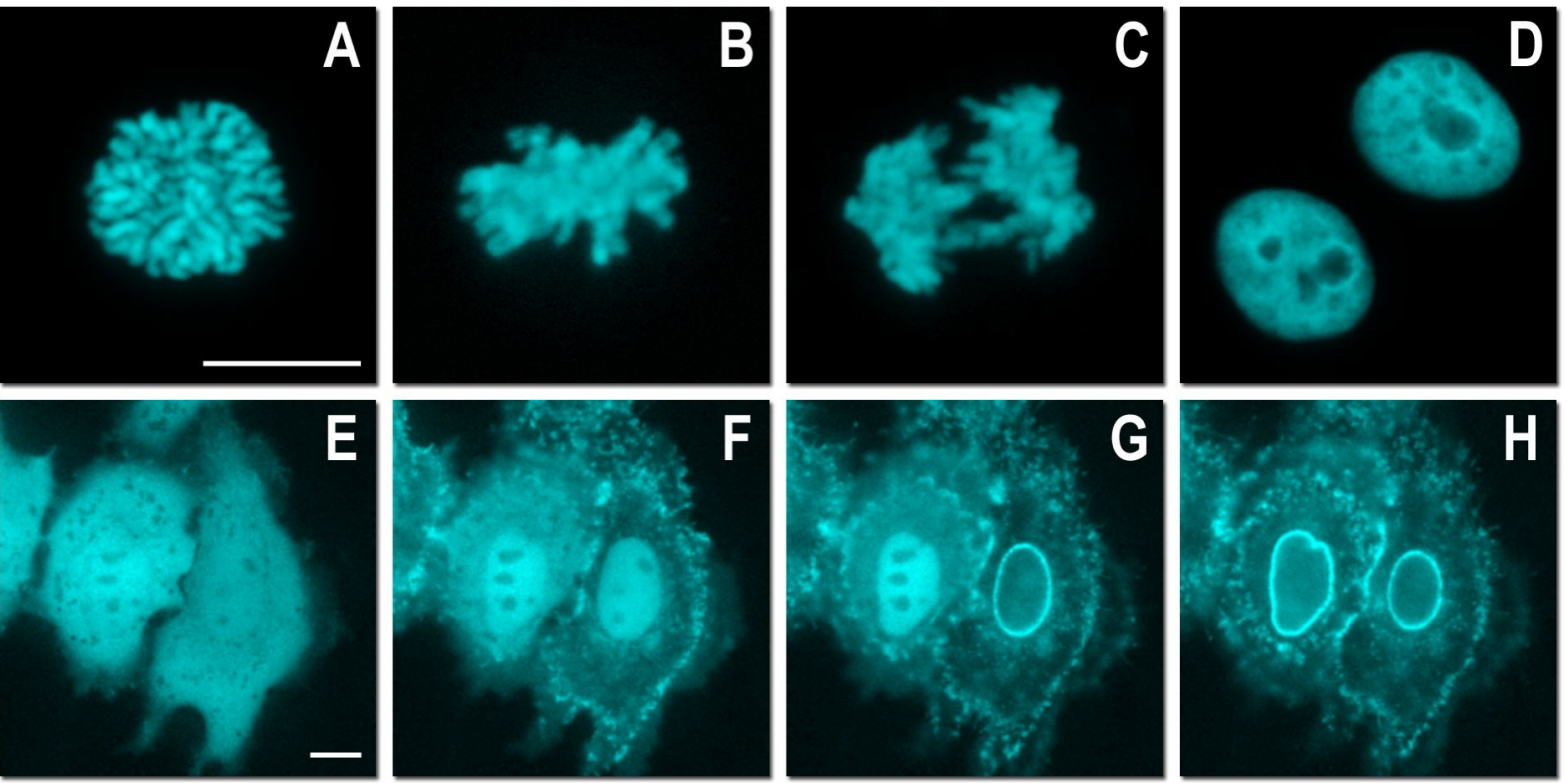
**Live cell imaging of mTFP1 fusion vectors**. (A)-(D) Laser scanning confocal images of a single HeLa cell expressing mTFP1-H2B-6 (N-terminus; human) progressing through prophase, metaphase, anaphase and interphase, respectively. (E)-(H) Spinning disk confocal images selected from a time-lapse series of HeLa cells expressing mTFP1-annexin (A4)-12 (C-terminus; human) during ionomycin-induced translocation to the plasma and nuclear membranes [35]: (E) 0 min, ionomycin added; (F) 5 min; (G) 7 min; (H) 9 min. Scale bars represent 10 μm.

**Figure 7:**
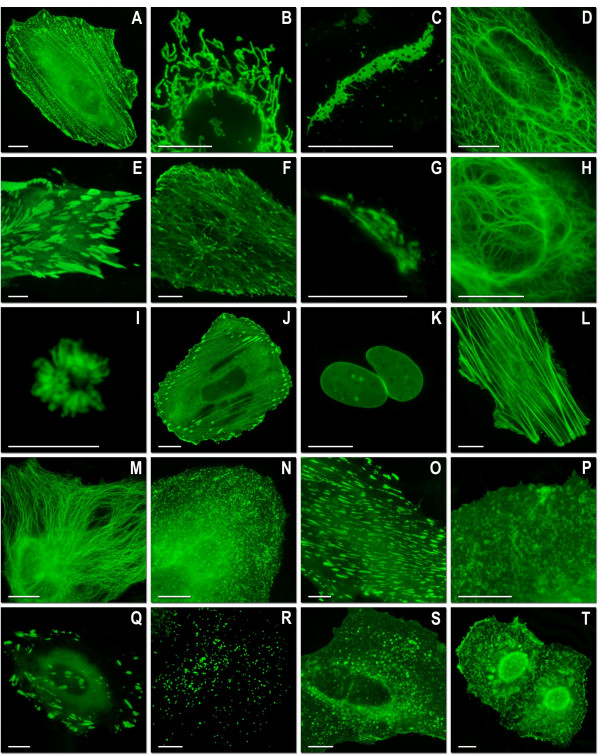
**Live cell imaging of mWasabi fusion vectors**. (A)-(J) N-terminal fusion constructs; for each fusion protein the linker amino acid length is indicated after the name of the targeted organelle or fusion protein: (A) mWasabi-α-actinin-19 (human non-muscle); (B) mWasabi-mitochondria-7 (human cytochrome C oxidase subunit VIII); (C) mWasabi-Cx26-7 (rat β-2 connexin-26); (D) mWasabi-keratin-17 (human cytokeratin 18); (E) mWasabi-paxillin-22 (chicken); (F) mWasabi-EB3-7 (human microtubule-associated protein; RP/EB family); (G) mWasabi-golgi-7 (N-terminal 81 amino acids of human β-1,4-galactosyltransferase); (H) mWasabi-vimentin-7 (human); (I)mWasabi-H2B-6 (human); (J) mWasabi-zyxin-7 (human). (K)-(T) C-terminal fusion constructs: (K) mWasabi-lamin B1-10 (human); (L) mWasabi-β-actin-7; (M) mWasabi-β-tubulin-6 (human); (N) mWasabi-clathrin light chain-15 (human); (O) mWasabi-vinculin-23 (human); (P) mWasabi-farnesyl-5 (20-amino acid farnesylation signal from c-Ha-Ras); (Q) mWasabi-focal adhesion kinase-5 (chicken protein tyrosine kinase 2); (R) mWasabi-peroxisomes-2 (peroximal targeting signal 1; PTS1); (S) mWasabi-endosomes-15 (human RhoB GTPase with an N-terminal c-Myc epitope tag); (T) mWasabi-annexin (A4)-15 (human). The cell line used for expressing mWasabi fusion vectors was gray fox lung fibroblast cells (FoLu) in panels (E) and (F) and human cervical adenocarcinoma cells (HeLa) in the remaining panels. Scale bars represent 10 μm.

In a previous paper we demonstrated that mTFP1 could be successfully fused with β-actin and α-tubulin protein without perturbing the native cytoskeletal structure [19]. In this work we sought to explore the range of proteins that would tolerate fusion to mTFP1 and mWasabi. We created a series of 22 different mTFP1 fusions (Figures 5 and 6) to both the C- and N-terminus of the FP and found that, in all cases, the fusion protein yielded a pattern of localization consistent with that observed for previously validated avGFP fusions. As shown in Figure 6, fusions to histone H2B and annexin A4 did not interfere with the normal cellular function of these proteins. A series of 20 similar fusions with mWasabi gave identical results (Figure 7). It is apparent that mTFP1 and mWasabi are robust and versatile FPs that tolerate a wide variety of protein fusions and subcellular microenvironments.

## Conclusion

In this manuscript we have described a series of mutagenesis experiments that have provided fundamental insight into the amino acids that dictate the color of the mTFP1 chromophore. Our data supports the conclusion that His163 and His197 act in an independent and additive fashion to increase the energy of the electronic transitions responsible for absorbance and fluorescence. Although determining the precise details of the mechanism are beyond the scope of this paper, our results are consistent with a previous proposal that electrostatic stabilization of charge density on the phenolate ring is a general means of achieving blue-shifted emission in FPs [20].

Our investigations into the amino acid determinants of the color of mTFP1 led us to a series of hue-shifted variants, one of which was subjected to further engineering and directed evolution to eventually produce mWasabi. While mWasabi is an exceptionally bright and reasonably photostable GFP, we readily acknowledge that for most experiments EGFP or Emerald should remain the GFP of choice. However, there are a number of specific applications, such as two-color imaging in conjunction with a Sapphire-type variant or as a FRET acceptor with a BFP donor, where the negligible excitation of mWasabi at 400 nm provides a substantial benefit. Both mTFP1 and mWasabi are well behaved in protein chimeras, offering a bright and photostable fluorescent signal with no significant perturbation of the localization or function of the protein of interest. This combination of desirable features firmly establishes mTFP1 and mWasabi as useful members of the FP toolkit.

## Methods

### General methods

Synthetic DNA oligonucleotides for cloning and library construction were purchased from Integrated DNA Technologies (Coralville, IA). PCR products and products of restriction digests were purified by gel electrophoresis and extraction using either the GenCatch™ gel extraction kit (Epoch Biolabs, TX) or the QIAquick™ gel extraction kit (QIAGEN, Valencia, CA). Plasmid DNA was purified from overnight cultures by using either the GeneJET™ Plasmid Miniprep Kit (Fermentas, ON) or the QIAprep Spin Miniprep kit (QIAGEN, Valencia, CA). Restriction endonucleases were purchased from either Invitrogen or New England Biolabs. Dye terminator cycle sequencing using the DYEnamic ET kit (Amersham Biosciences) was used to confirm the complete cDNA sequences for all FP variants and fusion constructs. Sequencing reactions were analyzed at the University of Alberta Molecular Biology Service Unit and the Florida State University Bioanalytical and Molecular Cloning DNA Sequencing Laboratory. All filters for fluorescence screening and imaging were purchased from Chroma Technology (Rockingham, VT), Omega Filters (Brattleboro, VT) and Semrock (Rochester, NY). The nucleotide sequence of mWasabi has been deposited in the GenBank^® ^nucleotide sequence database under accession number EU024648.

### Mutagenesis and library construction

Mutagenesis was performed by overlap PCR or error-prone PCR as described previously [19]. The PCR products were digested with Xho1 and EcoR1 and ligated into pBAD/His B vector digested with the same two enzymes (Invitrogen). The crude ligation mixture was used to transform electrocompetent *E. coli *strain DH10B (Invitrogen) which were then plated on Luria-Bertani (LB)/agar plates supplemented with ampicillin (0.1 mg/ml) and l-arabinose (0.02%). Plates were incubated for 14 h at 37°C prior to picking individual colonies (in the case of site-directed mutagenesis for creation of blue shifted variants) or fluorescence-based library screening for red shifted variants.

### Library screening

The screening system has been described previously [10] and is only described here in brief. The light from a 175 W xenon-arc lamp (Sutter) was passed through a bandpass filter selecting for 460–490 nm light. The light passed into a bifurcated fiber optic bundle (Newport) positioned to illuminate a Petri dish harboring bacterial colonies. The fluorescence emission of the colonies was screened by eye using tinted plastic goggles that block light with a wavelength of less than 500 nm. Colonies with more intense fluorescence were picked for further investigation. Colonies of interest were cultured overnight in 4 ml LB medium containing ampicillin (0.1 mg/ml) and l-arabinose (0.2%). The following day 0.1 ml of each culture was dispensed into individual wells of a clear bottom 96-well plate (Nunc) and the full emission spectra of each variant measured with a Safire2 plate reader equipped with monochromators (Tecan). Variants with the most intense and red-shifted fluorescence emission were used as templates in the subsequent round of library construction.

### Protein purification and characterization

For production of protein, *E. coli *strain LMG194 was transformed with the pBAD/His B expression vector containing the FP gene of interest. A single colony was used to inoculate a 4 ml culture that was allowed to grow overnight (37°C, 225 rpm) before being diluted into 1 l of LB medium supplemented with ampicillin and l-arabinose. The culture was grown for 12 h before cells were harvested by centrifugation and lysed by French Press. Proteins were purified by Ni-NTA chromatography (Amersham). Absorption spectra were recorded on a DU-800 UV-visible spectrophotometer (Beckman) and fluorescence excitation and emission spectra were recorded on a Safire2 plate reader. Reference standards for determining the quantum yields of BFP or GFP variants were quinine sulfate in 0.1 M H_2_SO_4 _or EGFP, respectively. Extinction coefficients were calculated using the protein concentration as determined by the bicinchoninic acid (BCA) method (Pierce) and the chromophore absorbance as determined by UV-visible spectroscopy. For fluorescence pKa measurements, the protein of interest was first dialyzed into dilute buffer (5 mM Tris HCl, pH 7.5) before being diluted into a series of 200 mM phosphate and imidazole buffers at various pH values. Fluorescence intensity was measured using a Safire2 plate reader.

### Photostability measurements

For photostability measurements of green-fluorescing variants, microdroplets of either the purified protein (100 μM) or *E. coli *culture (previously transformed with the expression plasmid and induced) was mixed with mineral oil and vortexed. Approximately 5 μl of this suspension was sandwiched between a glass slide and a glass cover slip. Individual drops were identified by fluorescence microscopy and subjected to photobleaching as previously described [10]. For all experiments, EGFP was subjected to bleaching under identical conditions and used as a reference standard.

### Mammalian expression vectors

To create the Sapphire-actin and mWasabi-NLS vectors, the genes encoding Sapphire (also known as H9-40) [2,13,33] and mWasabi were PCR amplified with a 5' primer encoding an NheI site and a 3' primer encoding an XhoI site. The purified and digested PCR products were ligated into pEGFP-actin or pEYFP-Nucleus (Clontech), respectively, which had been previously digested with the same restriction enzymes to excise the FP coding sequence. An analogous nuclear localization construct was made for EGFP. All of the other mTFP1 and mWasabi vectors were constructed using C1 and N1 (Clontech-style) cloning vectors. The FPs were amplified with a 5' primer encoding an AgeI site and a 3' primer encoding either a BspEI (C1) or Not1 (N1) site. The purified and digested PCR products were ligated into similarly digested EGFP-C1 and EGFP-N1 cloning vector backbones. To generate fusion vectors, the appropriate cloning vector and an EGFP fusion vector were digested, either sequentially or doubly, with the appropriate enzymes and ligated together after gel purification. Thus, to prepare mTFP1 and mWasabi N-terminal fusions, the following digests were performed: human non-muscle α-actinin, EcoRI and NotI (vector source, Tom Keller, FSU); human cytochrome C oxidase subunit VIII, BamHI and NotI (mitochondria, Clontech); human zyxin, BamHI and NotI (Clare Waterman-Storer, NIH); rat α-1 connexin-43 and rat β-2 connexin-26, EcoRI and BamHI (Matthias Falk, Lehigh University); human H2B, BamHI and NotI (George Patterson, NIH); N-terminal 81 amino acids of human β-1,4-galactosyltransferase, BamHI and NotI (Golgi, Clontech); human microtubule-associated protein EB3, BamHI and NotI (Lynne Cassimeris, Lehigh University); human vimentin, BamHI and NotI (Robert Goldman, Northwestern University); human keratin 18, EcoRI and NotI (Open Biosystems, Huntsville, AL); chicken paxillin, EcoRI and NotI (Alan Horwitz, University of Virginia); rat lysosomal membrane glycoprotein 1, AgeI and NheI (George Patterson, NIH); endoplasmic reticulum (calreticulin signal sequence and KDEL retention sequence), AgeI and EcoRI (Clontech). To prepare mTFP1 and mWasabi C-terminal fusions, the following digests were performed: human β-actin, NheI and BglII (Clontech); human α-tubulin, NheI and BglII (Clontech); human light chain clathrin, NheI and BglII (George Patterson, NIH); human lamin B1, NheI and BglII (George Patterson, NIH); human fibrillarin, AgeI and BglII (Evrogen); human vinculin, NheI and EcoRI (Open Biosystems, Huntsville, AL); peroximal targeting signal 1 (PTS1 – peroxisomes), AgeI and BspEI (Clontech); chicken protein tyrosine kinase 2, AgeI and BglII (Clare Waterman-Storer, NIH); human annexin (A4), AgeI and BspEI (Alen Piljic, EMBL, Heidelberg); human RhoB GTPase with an N-terminal c-Myc epitope tag (endosomes), AgeI and BspEI (Clontech); and the 20-amino acid farnesylation signal from c-Ha-Ras, AgeI and BspEI (membrane, Clontech). DNA for mammalian transfection was prepared by either the Plasmid Midi or Maxi kit (QIAGEN).

### Live cell imaging

HeLa epithelial (CCL-2, ATCC) and gray fox lung fibroblast (CCL-168, ATCC) cells were either cultured and transfected as described previously [19], or grown in a 50:50 mixture of DMEM and Ham's F12 with 12.5% Cosmic calf serum (Hyclone) and transfected with Effectene (QIAGEN). For dual-color imaging, the two expression plasmids were pre-mixed in a 1:1 ratio before transfection. Widefield live cell imaging was performed with a Zeiss Axiovert 200 M microscope equipped with appropriate filter sets (Chroma), a Nikon TE-2000 inverted microscope equipped with Omega filters, or an Olympus IX71 equipped with Semrock filters. Laser scanning confocal microscopy was conducted on a Nikon C1Si and an Olympus FV1000, both equipped with argon-ion 457 and 488 nm lasers and proprietary filter sets. Spinning disk confocal microscopy was performed on an Olympus DSU-IX81 equipped with a Lumen 200 illuminator (Prior, Boston, MA), Semrock filters, and 10-position filter wheels driven by a Lambda 10-3 controller (Sutter, Novato, CA).

Sapphire fluorescence was measured using a 375–415 nm bandpass excitation filter, a 475 nm longpass beamsplitter, and 500–550 nm bandpass emission filters. mTFP1 was imaged with a CFP filter set (96188, Nikon) or a custom set composed of a 430–460 nm bandpass excitation filter, a 475 nm longpass beamsplitter, and a 480–520 nm bandpass emission filter. EGFP and mWasabi were imaged using either a standard EGFP filter set (41017, Chroma), a QuantaMaxTM Green set (Omega), or a BrightLine GFP set (3035B, Semrock).

## Abbreviations

avGFP: *Aequorea victoria *GFP; BFP: blue FP; CFP: cyan FP; ECFP: enhanced CFP; EGFP: enhanced avGFP; FP: fluorescent protein; FRET: fluorescence resonance energy transfer; GFP: green FP; LB: Luria-Bertani; mTFP: monomeric teal FP; NLS: nuclear localization signal; PCR: polymerase chain reaction; YFP: yellow FP.

## Authors' contributions

H-wA carried out mutagenesis studies, protein characterization and two-color imaging experiments. PW assisted H-wA with the directed evolution of mWasabi. MWD and SGO carried out imaging of mWasabi and mTFP1 fusion proteins and assisted in preparation of the manuscript. REC conceived and designed the study and drafted the manuscript. All authors read and approved the final manuscript.

## References

[B1] Prasher DC, Eckenrode VK, Ward WW, Prendergast FG, Cormier MJ (1992). Primary structure of the *Aequorea victoria *green-fluorescent protein. Gene.

[B2] Tsien RY (1998). The green fluorescent protein. Annu Rev Biochem.

[B3] Chalfie M, Tu Y, Euskirchen G, Ward WW, Prasher DC (1994). Green fluorescent protein as a marker for gene expression. Science.

[B4] Inouye S, Tsuji FI (1994). Aequorea green fluorescent protein. Expression of the gene and fluorescence characteristics of the recombinant protein. FEBS Lett.

[B5] Wouters FS (2006). The physics and biology of fluorescence microscopy in the life sciences. Contemp Phys.

[B6] Lukyanov KA, Chudakov DM, Lukyanov S, Verkhusha VV (2005). Innovation: Photoactivatable fluorescent proteins. Nat Rev Mol Cell Biol.

[B7] Shaner NC, Steinbach PA, Tsien RY (2005). A guide to choosing fluorescent proteins. Nat Methods.

[B8] Betzig E, Patterson GH, Sougrat R, Lindwasser OW, Olenych S, Bonifacino JS, Davidson MW, Lippincott-Schwartz J, Hess HF (2006). Imaging intracellular fluorescent proteins at nanometer resolution. Science.

[B9] Zhang J, Campbell RE, Ting AY, Tsien RY (2002). Creating new fluorescent probes for cell biology. Nat Rev Mol Cell Biol.

[B10] Ai HW, Shaner NC, Cheng Z, Tsien RY, Campbell RE (2007). Exploration of new chromophore structures leads to the identification of improved blue fluorescent proteins. Biochemistry.

[B11] Rizzo MA, Springer GH, Granada B, Piston DW (2004). An improved cyan fluorescent protein variant useful for FRET. Nat Biotechnol.

[B12] Heim R, Cubitt AB, Tsien RY (1995). Improved green fluorescence. Nature.

[B13] Ehrig T, O'Kane DJ, Prendergast FG (1995). Green-fluorescent protein mutants with altered fluorescence excitation spectra. FEBS Lett.

[B14] Heim R, Tsien RY (1996). Engineering green fluorescent protein for improved brightness, longer wavelengths and fluorescence resonance energy transfer. Curr Biol.

[B15] Matz MV, Fradkov AF, Labas YA, Savitsky AP, Zaraisky AG, Markelov ML, Lukyanov SA (1999). Fluorescent proteins from nonbioluminescent Anthozoa species. Nat Biotechnol.

[B16] Matz MV, Lukyanov KA, Lukyanov SA (2002). Family of the green fluorescent protein: journey to the end of the rainbow. Bioessays.

[B17] Shaner NC, Campbell RE, Steinbach PA, Giepmans BN, Palmer AE, Tsien RY (2004). Improved monomeric red, orange and yellow fluorescent proteins derived from Discosoma sp. red fluorescent protein. Nat Biotechnol.

[B18] Shaner NC, Patterson GH, Davidson MW (2007). Advances in fluorescent protein technology. J Cell Sci.

[B19] Ai HW, Henderson JN, Remington SJ, Campbell RE (2006). Directed evolution of a monomeric, bright and photostable version of Clavularia cyan fluorescent protein: structural characterization and applications in fluorescence imaging. Biochem J.

[B20] Henderson JN, Remington SJ (2005). Crystal structures and mutational analysis of amFP486, a cyan fluorescent protein from *Anemonia majano*. Proc Natl Acad Sci USA.

[B21] Cinelli RA, Tozzini V, Pellegrini V, Beltram F, Cerullo G, Zavelani-Rossi M, De Silvestri S, Tyagi M, Giacca M (2001). Coherent dynamics of photoexcited green fluorescent proteins. Phys Rev Lett.

[B22] Marques MA, Lopez X, Varsano D, Castro A, Rubio A (2003). Time-dependent density-functional approach for biological chromophores: the case of the green fluorescent protein. Phys Rev Lett.

[B23] Gurskaya NG, Savitsky AP, Yanushevich YG, Lukyanov SA, Lukyanov KA (2001). Color transitions in coral's fluorescent proteins by site-directed mutagenesis. BMC Biochem.

[B24] Budisa N, Pal PP, Alefelder S, Birle P, Krywcun T, Rubini M, Wenger W, Bae JH, Steiner T (2004). Probing the role of tryptophans in *Aequorea victoria *green fluorescent proteins with an expanded genetic code. Biol Chem.

[B25] Patterson GH, Lippincott-Schwartz J (2002). A photoactivatable GFP for selective photolabeling of proteins and cells. Science.

[B26] Ormo M, Cubitt AB, Kallio K, Gross LA, Tsien RY, Remington SJ (1996). Crystal structure of the *Aequorea victoria *green fluorescent protein. Science.

[B27] Cormack BP, Valdivia RH, Falkow S (1996). FACS-optimized mutants of the green fluorescent protein (GFP). Gene.

[B28] Ward WW, Cormier MJ (1979). An energy transfer protein in coelenterate bioluminescence. Characterization of the Renilla green-fluorescent protein. J Biol Chem.

[B29] Karasawa S, Araki T, Yamamoto-Hino M, Miyawaki A (2003). A green-emitting fluorescent protein from Galaxeidae coral and its monomeric version for use in fluorescent labeling. J Biol Chem.

[B30] Cubitt AB, Woollenweber LA, Heim R (1999). Understanding structure-function relationships in the *Aequorea victoria *green fluorescent protein. Methods Cell Biol.

[B31] Waldo GS, Standish BM, Berendzen J, Terwilliger TC (1999). Rapid protein-folding assay using green fluorescent protein. Nat Biotechnol.

[B32] Pedelacq JD, Cabantous S, Tran T, Terwilliger TC, Waldo GS (2006). Engineering and characterization of a superfolder green fluorescent protein. Nat Biotechnol.

[B33] Heim R, Prasher DC, Tsien RY (1994). Wavelength mutations and posttranslational autoxidation of green fluorescent protein. Proc Natl Acad Sci USA.

[B34] Mitra RD, Silva CM, Youvan DC (1996). Fluorescence resonance energy transfer between blue-emitting and red-shifted excitation derivatives of the green fluorescent protein. Gene.

[B35] Piljic A, Schultz C (2006). Annexin A4 self-association modulates general membrane protein mobility in living cells. Mol Biol Cell.

